# Tooth loss and oral health-related quality of life: a systematic review and meta-analysis

**DOI:** 10.1186/1477-7525-8-126

**Published:** 2010-11-05

**Authors:** Anneloes E Gerritsen, P Finbarr Allen, Dick J Witter, Ewald M Bronkhorst, Nico HJ Creugers

**Affiliations:** 1 Department of Oral Function and Prosthetic Dentistry, College of Dental Science, Radboud University Nijmegen Medical Centre, Philips van Leydenlaan 25, 6525 EX Nijmegen, The Netherlands; 2 Department of Restorative Dentistry, University Dental School & Hospital, Wilton, Cork, Ireland; 3 Department of Community and Restorative Dentistry, College of Dental Science, Radboud University Nijmegen Medical Centre, Philips van Leydenlaan 25, 6525 EX Nijmegen, The Netherlands

## Abstract

**Background:**

It is increasingly recognized that the impact of disease on quality of life should be taken into account when assessing health status. It is likely that tooth loss, in most cases being a consequence of oral diseases, affects Oral Health-Related Quality of Life (OHRQoL). The aim of the present study is to systematically review the literature and to analyse the relationship between the number and location of missing teeth and oral health-related quality of life (OHRQoL). It was hypothesized that tooth loss is associated with an impairment of OHRQoL. Secondly, it was hypothesized that location and distribution of remaining teeth play an important role in this.

**Methods:**

Relevant databases were searched for papers in English, published from 1990 to July 2009 following a broad search strategy. Relevant papers were selected by two independent readers using predefined exclusion criteria, firstly on the basis of abstracts, secondly by assessing full-text papers. Selected studies were grouped on the basis of OHRQoL instruments used and assessed for feasibility for quantitative synthesis. Comparable outcomes were subjected to meta-analysis; remaining outcomes were subjected to a qualitative synthesis only.

**Results:**

From a total of 924 references, 35 were eligible for synthesis (inter-reader agreement abstracts κ = 0.84 ± 0.03; full-texts: κ = 0.68 ± 0.06). Meta-analysis was feasible for 10 studies reporting on 13 different samples, resulting in 6 separate analyses. All studies showed that tooth loss is associated with unfavourable OHRQoL scores, independent of study location and OHRQoL instrument used. Qualitative synthesis showed that all 9 studies investigating a possible relationship between number of occluding pairs of teeth present and OHRQoL reported significant positive correlations. Five studies presented separate data regarding OHRQoL and location of tooth loss (anterior tooth loss *vs*. posterior tooth loss). Four of these reported highest impact for anterior tooth loss; one study indicated a similar impact for both locations of tooth loss.

**Conclusions:**

This study provides fairly strong evidence that tooth loss is associated with impairment of OHRQoL and location and distribution of tooth loss affect the severity of the impairment. This association seems to be independent from the OHRQoL instrument used and context of the included samples.

## Background

It is increasingly recognized that the impact on quality of life (QoL) of disease and treatment of disease and its consequences should be taken into account when assessing health status and evaluating treatment outcomes. Clinical indicators only are not sufficient to describe health status and it has been reported that people with chronic disabling disorders can perceive their quality of life as better than healthy individuals, i.e., poor health or presence of disease does not inevitably mean poor quality of life [1,2]. Adaptive capacity and personal characteristics appear to influence patient's response to chronic disease. This can result in reports which seem counterintuitive, for example, the finding in a large German survey that having fewer than 9 teeth had more impact on health-related QoL than having cancer, hypertension, or allergy [3]. Therefore, clinical indicators only are not sufficient to describe health status. This is also true for oral diseases and its consequences for oral health-related quality of life (OHRQoL). The two most prevalent oral diseases, caries and periodontal disease often do not cause symptoms in early stages. This might explain that clinical indicators of caries or periodontal involvement, such as number of decayed teeth, respectively tooth mobility and pocket depth are not strongly associated with impairment of OHRQoL [4,5]. However, caries and periodontal disease are progressive processes, and lead to tooth loss if not treated adequately. Tooth loss will presumably cause functional impairment, for example, with regard to chewing and esthetics, depending on the location of tooth loss, which might ultimately affect QoL.

Besides generic health related QoL measures, specific oral health-related quality of life models and measures have been developed to assess the impact of oral disease on OHRQoL [6]. For example Locker [7] described a model based on the WHO classification of impairment, disability and handicap. The Oral Health Impact Profile (OHIP), one of the most popular measures, was developed on basis of this model [8].

Although OHRQoL assessment by validated questionnaires is more common nowadays, a recent systematic review of the literature resulted in only sparse information regarding OHRQoL treatment outcomes of reconstructive dentistry for partially edentate patients [9]. However, besides using OHRQoL measures to evaluate treatment outcomes it is in the first place important to know to what extent tooth loss actually affects OHRQoL. This enables development of clinical decision making in public health and to provide appropriate oral health care. Several population surveys include 'number of teeth' in statistical models analyzing impact on OHRQoL, but this parameter appears not always to be the most prominent predictor. For example, in a population of older adults in Sri Lanka, Ekanayake [10] found only a weak association between tooth loss and other clinical parameters on the one hand and oral impacts on the other hand. This suggests that other factors such as age, gender or cultural background of the patient play an important role in the perception of health [10,11]. In contrast, in a large Japanese study Ide et al [12] found a strong correlation between the number of missing teeth and higher OHIP scores suggesting impairment of OHRQoL.

The aim of the present study is to systematically review the literature and to analyse the relationship between the number and location of missing teeth and oral health-related quality of life (OHRQoL). It was hypothesized that tooth loss is associated with an impairment of OHRQoL. Secondly, it was hypothesized that location and distribution of remaining teeth play an important role in this.

## Methods

### Search strategy

In this study the Cochrane guidelines for the conduct of a systematic review were used [13]. Medline, PubMed, Embase and the Cochrane Library were initially searched for papers published from 1990 to June 2008 to answer the following question: is tooth loss associated with impairment of people's oral health related quality of life and what is the role of location and distribution of tooth loss in this relationship? The search was updated in July 2009. A broad search strategy was pursued to capture as many relevant studies as possible. For this reason not only studies with subject matter 'tooth loss' but also studies with subject matter 'management of tooth loss' were searched for. The following keywords were used: 'quality of life', 'patient satisfaction', 'tooth loss', 'partial edent*', 'partial denture', 'implant' and 'prosthodont*'. MeSH terms were used if the search machine of the database permitted this. The full search strategy for PubMed is presented in Table 1. As in the early nineteen nineties quality of life was not a general used concept in dentistry, patient satisfaction was used as a proxy of quality of life. Although RCT's provide the highest level of evidence, this study design is in most cases not feasible for tooth loss. Therefore, data from observational studies like cross-sectional studies, case series, case-control and cohort studies are included in this review [14]. Only publications in English were selected. Reference lists of the eventually included papers were hand-searched to identify additional relevant studies and possible false exclusions, until no new applicable titles appeared (saturation).

**Table 1:** PubMed search as used

#1	("Quality of Life"[MeSH]) OR ("Patient Satisfaction"[MeSH])
#2	("Denture, Partial"[MeSH]) OR ("Denture, Partial, Fixed"[MeSH]) OR ("Dental Implants"[MeSH]) OR ("Dental Implants, Single-Tooth"[MeSH]) OR ("Dental Prosthesis, Implant-Supported"[MeSH]) OR ("Osseointegration"[MeSH]) OR ("Dental Implantation"[MeSH])

#3	("Jaw, Edentulous, Partially"[MeSH]) OR ("Tooth Loss"[MeSH])

#4	(#1 AND #2)

#5	(#1 AND #3)

#6	(#4 OR #5)

PubMed search using MeSH terms

### Study selection

Two readers (NHJC and AEG) independently selected references on the basis of titles and abstracts for the impact of tooth loss or tooth replacement on oral health-related quality of life using predefined exclusion criteria. Excluded were case reports, (narrative) reviews, non-human studies, non-oral implants (hip/knee) studies, studies exclusively dealing with edentulous subjects/full (over)dentures, restorations not replacing teeth, orthodontics, periodontics, tooth wear, and medical compromised patient groups (e.g. irradiated patients and systemic diseases like diabetes). The readers were calibrated by discussion sessions after assessment of every 10 abstracts. If necessary, the list of excluding criteria was revised after a discussion session and those abstracts already screened were re-subjected to the selection process. This procedure was repeated until no new exclusion criteria turned up. Agreement between readers was determined using κ statistics. Disagreements were resolved by discussion and if not resolved a third reader was called in (PFA) and reviewed the manuscript independently. In cases of doubt, the reference was included. This approach was applied in all selection steps.

After abstract selection, full-text copies of the selected papers were made. These full-text papers were assessed independently by the two readers (NHJC and AEG) using a pilot-tested assessment form. Full-text paper exclusion criteria are outlined in Table 2. In this phase of the review process, if considered necessary, authors were contacted to clarify issues of their published research that gave rise to uncertainty.

**Table 2:** Exclusion criteria applied for eligibility assessment of full-text papers and number of exclusions

Reason for exclusion (eligibility)	Number of studies excluded
Incomplete sample information	10
• Sampling method unclear	
• Age distribution not stated	
• Gender distribution not stated	
Insufficient methods (information)	26
• No clinical examination or validated 'self tooth count' form not used	
• Measure for satisfaction or OHRQoL not clearly described	
• Details of replacement not explicit	
OHRQoL outcomes not related to (management of) tooth loss	73
Mistakenly included on the basis of abstract	20
Total	129

### Synthesis of data

Studies were grouped on the basis of OHRQoL instruments used: Oral Health Impact Profile (OHIP), Oral Impact on Daily Living (OIDP), Geriatric Oral Health Assessment Index (GOHAI), Dental Impact of Daily Living (DIDL), OHQoL-UK^©^, and others. The rationale for this grouping was the incompatibility of the various instrument scoring systems. Besides that, the categorizations of number of teeth as used in the original studies should be comparable. Subsequently, for studies presenting continuous outcomes (e.g. mean scores) meta-analysis was deemed possible if a variance estimate was presented such as SD or SE. For studies presenting dichotomized outcomes pooling was considered possible if numbers with or without outcome property (e.g. with or without impact) were presented.

For continuous data Cochran's Q [15] was calculated to test for heterogeneity. Summary effects were calculated with DerSimonian's method [16] in case of heterogeneous data and weighted average was calculated for homogeneous data.

For dichotomized data Woolf's test [17] for heterogeneity was used. Again, summary effects were calculated by DerSimonian's method [16] in case of heterogeneous data, but the Mantel-Haenszel test [18] was used for homogenous data.

All studies, including those not suitable for meta-analyses, were subjected to qualitative analyses. For qualitative analyses study characteristics, main outcomes concerning missing teeth and possible other relevant outcomes were extracted and grouped according to OHRQoL instrument used.

## Results

### Study selection and study characteristics

**Figure 1:**
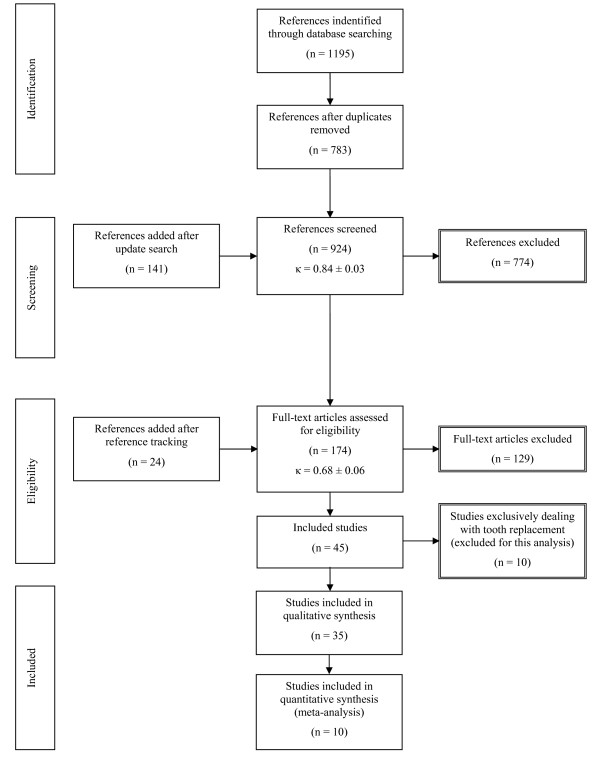
**Flow chart outlining the search strategy and results along various steps**.

Details of the identification, screening and selection process are presented in Figure 1. A total of 396 references was identified through the searching of Medline, 516 through PubMed, 134 through Embase, and 149 through the Cochrane Library. Duplicate references were removed and eventually 783 references remained. The search update resulted in 141 additional abstracts. For abstract assessment complete agreement was seen for 884 abstracts (inter-reader agreement κ = 0.84; SE = 0.03) and consensus was reached in 40 cases (23 included, 17 excluded). After reviewing the abstracts, 150 studies were included in the study. Reference tracking revealed 24 additional papers adding up to a total of 174 full-text papers for eligibility assessment. Finally, after assessment of full-text articles, 45 papers were included for review (inter-reader agreement κ = 0.68; SE = 0.06). In 5 cases the third reader's judgement was decisive. As the present study is only dealing with tooth loss, and not with management of tooth loss, studies exclusively dealing with the latter were not used for the present analyses. Characteristics and main outcomes of the 35 remaining studies [4,5,10,12,19-49] are presented in Additional file 1, Table S1; a summary of the data and feasibility for meta-analysis are presented in Additional file 2, Table S2.

### Quantitative analyses

**Figure 2:**
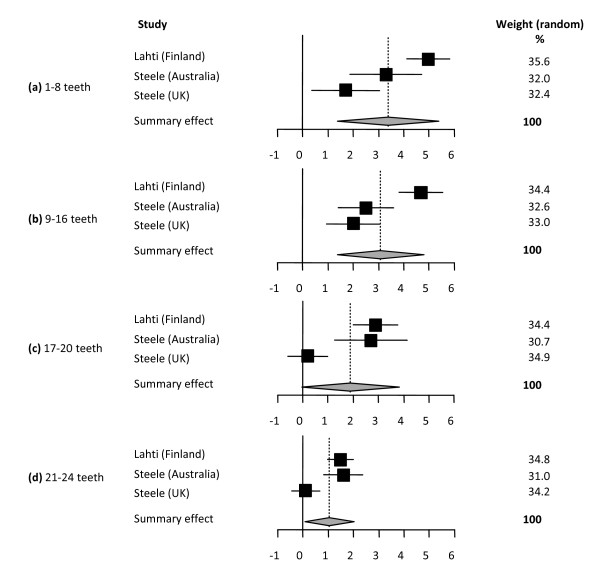
**Synthesis of two studies presenting differences in mean OHIP total scores**. Forest plots presenting differences in mean OHIP total scores of categories of number of present teeth for three samples (total n = 12,965) [25,43]. The category 25-32 teeth was used as reference. Relative box size indicates the weight of the study: **(a) **1-8 teeth (heterogeneity Q = 16.75; df = 2), **(b) **9-16 teeth (heterogeneity Q = 17.80; df = 2), **(c) **17-20 teeth (heterogeneity Q = 22.06; df = 2), **(d) **21-24 teeth (heterogeneity Q = 15.51; df = 2).

**Figure 3:**
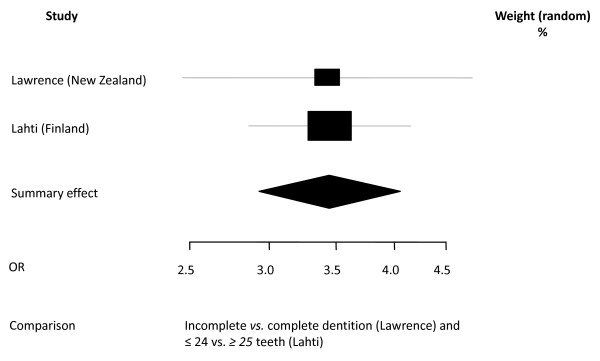
**Synthesis of two OHIP studies presenting Odds Ratio's**. Forest plot presenting Odss Ratio's (OR) for having OHIP impacts (fairly/very often) of two categories of number of present teeth (incomplete *vs*. complete [26] and ≤ 24 *vs*. ≥ 25 [25]) in two samples (total n = 6821). Relative box size indicates weight of the study (heterogeneity Χ^2 ^= 0,00; df = 1).

**Figure 4:**
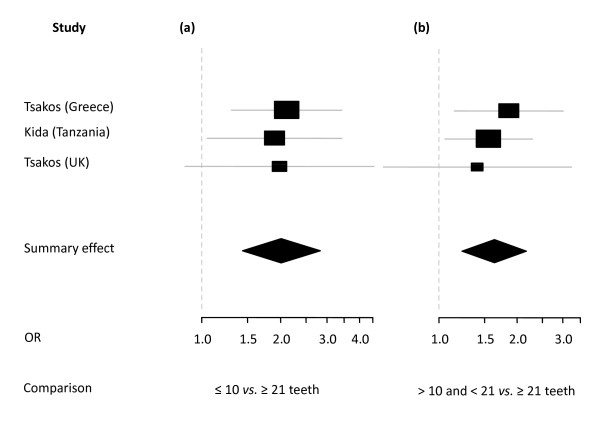
**Synthesis of three OIDP studies presenting Odds Ratio's**. Forest plots presenting Odss Ratio's (OR) for having any impact on OIDP of three categories of number of present teeth in three samples (total n = 2204) [5,24,45]. Relative box size indicates weight of the study. **(a) **≤ 10 *vs*. ≥ 21 (heterogeneity Χ^2 ^= 0.08; df = 2), **(b) **>10 and < 21 *vs*. ≥ 21 teeth (heterogeneity Χ^2 ^= 0.46; df = 2).

**Figure 5:**
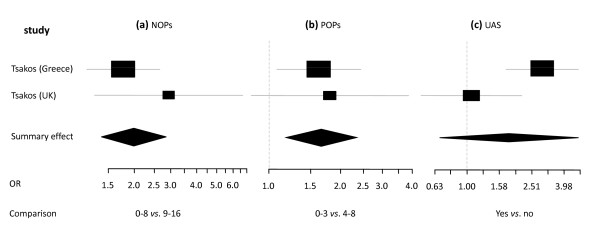
**Synthesis of two OIDP studies presenting Odds Ratio's in relation to occluding pairs and location**. Forest plots presenting Odss Ratio's (OR) for having any impact on OIDP of two categories of number of natural occluding pairs (NOPs) and posterior occluding pairs (POPs) and unrestored anterior spaces (UAS) in two samples (total n = 1184) [5,45]. Relative box size indicates weight of the study. **(a) **NOPs 0-8 *vs. *9-16 (heterogeneity Χ^2 ^= 1.17; df = 1), **(b) **POPs 0-3 *vs*. 4-8 (heterogeneity Χ^2 ^= 0.06; df = 1), **(c) **UAS yes *vs*. no (heterogeneity Χ^2 ^= 5.03; df = 1).

**Figure 6:**
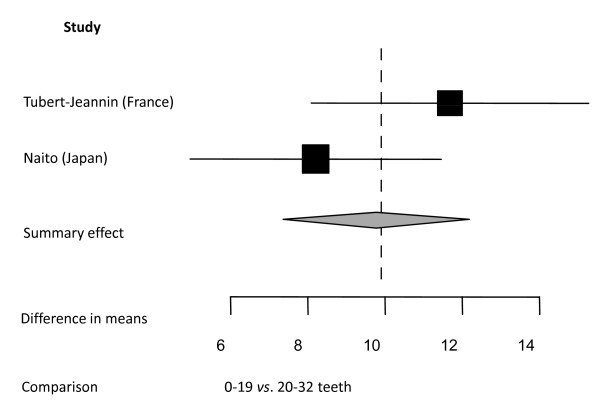
**Synthesis of two studies presenting differences in mean GOHAI total scores**. Forest plot presenting differences in mean GOHAI total scores between two categories of number of present teeth: 0-19 teeth *vs*. 20+ teeth in two samples (total n = 435) [38,46]. Relative box size indicates weight of the study (heterogeneity Q = 2.00; df = 1).

**Figure 7:**
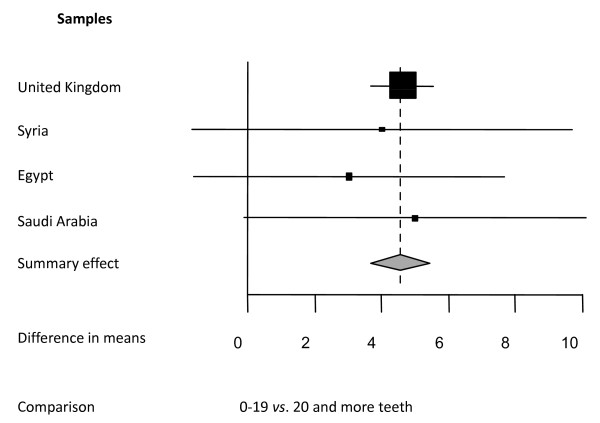
**Synthesis of two studies presenting differences in mean OHQoL-UK^© ^total scores in four samples**. Forest plot presenting differences in mean OHQoL-UK^© ^total scores between two categories of numbers of present teeth: 0-19 teeth *vs*. 20 and more teeth in four samples (total n = 2738) described in two studies [31,35]. Relative box size indicates weight of the study (heterogeneity Q = 0.15; df = 3).

In summary, 10 studies reporting on 13 different samples were eligible for meta-analysis resulting in 6 separate syntheses on the outcomes of 4 OHRQoL instruments (Table 3, Figures 2, 3, 4, 5, 6, 7).

**Table 3:** Summary of the 6 meta-analyses

Comparison	Summary effect	95% CI	p-value for heterogeneity test	Model used
***Meta analysis 1 ***[25,43] *Continuous data (difference in mean OHIP total scores)* *Total n = 12,965* *Reference group: 25-32 teeth*				
1-8 teeth	3.37	1.37-5.38	<0.001	random effect
9-16 teeth	3.08	1.37-4.80	<0.001	random effect
17-20 teeth	1.89	-0.03-3.82	<0.001	random effect
21-24 teeth	1.05	0.07-2.02	<0.001	random effect

***Meta analysis 2 ***[25,26] *Dichotomized data (Odds ratio for having an OHIP impact)* *Total n = 6821* *Reference group: complete dentition or ≥ 25 teeth*				
Incomplete or < 25 teeth	3.45	2.93-4.05	0.975	fixed effect

***Meta analysis 3 ***[5,24,45] *Dichotomized data (Odds ratio for having an OIDP impact)* *Total n = 2204* *Reference group ≥ 21 teeth*				
≤ 10 teeth	2.01	1.43-2.83	0.962	fixed effect
>10 and <21 teeth	1.63	1.23-2.17	0.794	fixed effect

***Meta analysis 4 ***[5,45] *Dichotomized data (Odds ratio for having an OIDP impact)* *Total n = 1184* *Reference groups 9-16 NOPs/4-8 POPs/no UAS*				
0-8 NOPs	1.99	1.39-2.86	0.279	fixed effect
0-3 POPs	1.66	1.16-2.37	0.808	fixed effect
UAS	1.82	0.68-4.87	0.025	random effect

***Meta analysis 5 ***[38,46] *Continuous data (difference in mean GOHAI total scores)* *Total n = 435* *Reference group: 20-32 teeth*				
0-19 teeth	9.78	7.38-12.18	0.157	fixed effect

***Meta analysis 6 ***[31,35] *Continuous data (difference in mean OHQoL-UK total scores)* *Total n = 2738* *Reference group: 20-32 teeth*				
0-19 teeth	4.56	3.67-5.44	0.912	fixed effect

#### Oral Health Impact Profile (OHIP) studies

Two studies [25,43] reported OHIP data as mean total scores (SD) from three different samples of three cross-sectional surveys from the UK (n = 3662), Australia (n = 3406) and Finland (n = 5987). In this analyses mean OHIP scores of subjects with 25-32 teeth were compared with mean OHIP scores of subjects having 21-24 teeth, 17-20 teeth, 9-16 teeth and 1-8 teeth (Figure 2). Data are presented as differences in mean OHIP scores per group for each sample. This meta-analysis shows that the fewer teeth are present the higher the impact on OHRQoL with a marked deterioration once the number of remaining teeth drops below 17.

Two studies [25,26], including in total 6,821 subjects, reported OHIP data as prevalence of impacts according to dental status (Figure 3). One study categorized dental status as complete dentition *vs*. one or more missing teeth [26] whereas the other study categorized dental status as 32-25 teeth *vs*. 0-24 teeth [25]. This pooling was made on the assumption that categories were comparable. Differences in impact scores between the two categories in each study are presented as Odds Ratios. The pooled data indicate that loss of teeth is associated with a threefold likelihood of reporting an impact on OHRQoL.

#### Oral Impact on Daily Living (OIDP) studies

The three studies [5,24,45], including in total 2204 subjects, that used OIDP scores as an outcome measure for OHRQoL are presented in Figure 4. In all three studies OIPD scores were calculated by multiplying frequency by severity of the impact and summing up the scores of ten areas of daily activities. Three categories of dental status were presented namely 0/1-10, 11-20 and 21-32 teeth present. Differences between the categories were presented as Odds Ratios with having an impact as dependent variable. Subjects with fewer than 10 teeth were twice as likely to report an impact compared with subjects having 21-32 teeth; subject with 11-20 teeth were 1.5 times more likely to report an impact.

Two of the OIDP studies [5,45] (total number of subjects = 1184) presented OHRQoL data in relation to occluding pairs and location of tooth loss: natural occluding pairs (NOPs), posterior occluding pairs (POPs), and presence of 'unrestored anterior spaces' (UAS). Differences between categories are presented as Odds Ratio's with having an impact as dependent variable (Figure 5). Reporting an impact on their daily life was twice as likely for subjects with 0-8 NOPs than for subjects having 9-16 NOPs and 1.6 times more likely for subjects having 0-3 POPs than for subjects having 4-8 POPs. Subjects having one or more unrestored anterior spaces were 1.8 times more likely to report any impact on their daily life.

#### Geriatric Oral Health Assessment Index (GOHAI) studies

The two GOHAI studies [38,46] (total n = 435) in this meta-analysis used mean total scores as outcome measure (Figure 6). Differences in the mean scores show that GOHAI scores were higher for subjects with 20 or more teeth, indicating better OHRQoL.

#### Oral Health Quality of Life-UK (OHQoL-UK^© ^)studies

Two studies [31,35] reported mean total scores for OHQoL-UK^© ^for four different samples from the UK, Syria, Egypt and Saudi Arabia with a total of 2783 subjects (Figure 7). Differences between mean total scores of two categories of dental status, namely 0-19 teeth present *vs*. 20 and more teeth. It should be noted that the UK sample contributes 91% to the summary effect.

### Qualitative analyses

The studies that failed the criteria for meta-analysis were only analyzed qualitatively.

#### Number of teeth

Most included studies found statistically significant associations between missing teeth and unfavourable OHQoL scores, independent of the instrument used or the country of investigation. However, the results of a few studies were not conclusive: Hassel [23] reported no statistically significant difference in OHIP scores between dentate and edentate institutionalized elderly, but statistically significant higher OHIP scores for subject with 'less teeth in static occlusion'; Mesas [37] reported only statistically significant differences in GOHAI scores between edentulous and dentate subjects for the 'physical' dimension but not for the 'social' and 'worry' dimension; Tsakos [5] and Sheiham [41], reporting on the same sample, found no statistically significant association between number of present teeth and having an OIPD impact in British adults, but lower numbers of anterior occluding pairs and natural occluding pairs were associated with OHRQoL impairment.

#### Occluding pairs and location of missing teeth

Statistically significant positive correlations between number of occluding pairs and OHRQoL were found in all 10 studies (dealing with 9 different samples) reporting on this subject [5,20,21,23,27,29,36,37,44,45] (Table 4).

**Table 4:** Summary of studies reporting on occluding pairs

First author, year	Population, sample n, (% females)	Subject of the study	Main outcomes regarding occluding pairs
*OHIP-49 (Oral Health Impact Profile)*			

Baba, 2008a^CS ^[20] Baba, 2008b^CS ^[21]	Japanese adults with shortened dental arches n = 155 (70)	Relationship between shortened dental arches and OHRQoL	a: Dose response relationship between number of missing posterior teeth and OHRQoL in subjects with shortened dental arches. Missing posterior units is related to impairment of OHRQoL. b: Patterns of missing occluding units likely to be related to the OHRQoL impairment in shortened dental arch subjects with the presence of first molar contact having a particularly important role.

Hassel,2006^CS ^[23]	German institutionalized elderly n = 159 (81)	Dental and non-dental factors on OHRQoL of institutionalized elderly	Less teeth in static occlusion related to impairment of OHRQoL.

Locker, 1994^LT ^[29]	Canadian older adults n = 312 (54)	Clinical and subjective indicators of oral health status and OHRQoL	Having fewer functional units associated with impairment of OHRQoL.

*GOHAI (Geriatric Oral Health Assessment Index)*			

Mesas, 2008^CS ^[37]	Brazilian urban elderly n = 267 (60)	Dental and non-dental factors on OHRQoL	Absence of posterior occlusion associated with impairment of OHRQoL but only statistically significant for 'physical' dimension and not for the 'social' and 'worry' dimensions.

Swoboda, 2006^CS ^[44]	American low income elderly n = 733 (56)	Dental and non-dental predictors on OHRQoL	OHRQoL positively related to the total number of occluding pairs, molar pairs occluding, anterior pairs occluding, and premolar pairs occluding.

*OIDP (Oral Impact on Daily Performance)*			

Tsakos, 2006^CS ^[5]	British non-institutionalized elderly (subsample of Sheiham, 2001) n = 736 (48)	Clinical correlates of OHRQoL	OHRQoL significantly related to the total number of occluding pairs and to the number of anterior occluding pairs but not to the number of posterior occluding pairs.

Tsakos, 2004^CS ^[45]	Greek non-institutionalized elderly n = 448 (64)	Relationship between clinical dental measures and OHRQoL	OHRQoL significantly related to the total number of occluding pairs and to the number of posterior occluding pairs.

*Ad hoc satisfaction questionnaires*			

Leake, 1994^CS ^[27]	American and Canadian older adults n = 338 (55)	Assessment of relationship between oral function and posterior dental units	Low number of posterior units was associated with embarrassment and dissatisfaction on chewing and appearance, indicating OHRQoL impairment.

Meeuwissen, 1995^CS ^[36]	Dutch dentate older adults n = 320 (59)	Satisfaction with reduced dentitions	Fewer posterior occluding units associated with lower satisfaction scores, indicating OHRQoL impairment.

^CS ^= cross-sectional study; ^LT ^= longitudinal study; ^CO ^= cohort study; ^VA ^= validation study

Five studies reported on OHRQoL and location of missing teeth, four of them [5,40,45,48] reporting higher impact for missing anterior teeth. One of them [44] indicated comparable impact for missing posterior occluding pairs and anterior occluding pairs (Table 5).

**Table 5:** Summary of studies reporting the location of missing teeth

First author, year	Population, sample n, (% females)	Subject of the study	Main outcomes regarding location of missing teeth
*OHIP-49 (Oral Health Impact Profile)*			

Walter, 2007^CS ^[48]	Canadian rural adults n = 140 (64)	Clinical and socio-demographic variables and OHRQoL	One or more natural posterior teeth missing not associated with OHRQoL impairment whereas one or more natural anterior teeth missing was associated with OHRQoL impairment.

*OHIP-14 (Oral Health Impact Profile short version)*			

Pallegedara, 2008^CS ^[40]	Sinhalese non-institutionalized elderly n = 630 (54)	Tooth loss, denture status and OHRQoL	'Presence of anterior spaces' more negative impact on the OHRQoL than 'missing posterior teeth'.

*GOHAI (Geriatric Oral Health Assessment Index)*			

Swoboda, 2006^CS ^[44]	American low income elderly n = 733 (56)	Dental and non-dental predictors on OHRQoL	Comparable impact on OHRQoL of the number of molar pairs occluding, premolar pairs occluding and anterior pairs occluding.

*OIDP (Oral Impact on Daily Performance)*			

Tsakos, 2004^CS ^[45]	Greek non-institutionalized elderly n = 448 (48)	Relationship between clinical dental measures and OHRQoL	Having 'unfilled anterior spaces' more impact on OHRQoL than having few (0-3) posterior occluding pairs.

Tsakos, 2006^CS ^[5]	British non-institutionalized elderly n = 736 (64)	Clinical correlates of OHRQoL	Having few anterior occluding pairs (0-2) more impact on OHRQoL than having few posterior occluding pairs (0-3).

^CS ^= cross-sectional study; ^LT ^= longitudinal study; ^CO ^= cohort study; ^VA ^= validation study

## Discussion

Chronic diseases such as dental caries are still highly prevalent in older adults, and the risk of tooth loss in old age is high. Oral health care with an intervention led focus is costly, and demand for this care may increase as the proportion of older dentate adults increases. Demand for treatment is not well correlated with objectively determined treatment need, and it has been recognized that objective measures of disease are not good predictors of demand. It would appear that loss of teeth is not as acceptable as in previous generations, and this will potentially influence future demand for treatment [50]. As public resources for dental treatment becomes increasingly scarce, new paradigms for assessment of oral health have been developed. The use of OHRQoL measures has increased significantly over the past 15 years. By incorporating subjective and objective assessment, our understanding of the consequences of oral disease and tooth loss has improved [51]. Subjective assessment has also been advocated as a means of targeting treatment resources provided through publically funded health services [52]. The rationale for this is to prioritise scarce financial resources towards those eligible patients most likely to benefit from a particular therapy. It is known that the impact of disease on quality of life is highly variable, and thus, the impact of a treatment intervention will also vary. An example of this is in the use of dental implants to retain prostheses in edentulous patients. Dental status (in this case, edentate) does not necessarily predict treatment outcome, and edentate patients satisfied with having complete dentures are unlikely to report significant extra benefit from having an expensive intervention (e.g., implant retained dentures) [53]. In this scenario, a health service provider would prefer to target resources towards patients who are dissatisfied with being edentate and have a poor self-reported health status. This is particularly relevant where a cure is not the objective of treatment, and the treatment goal is a reduction in morbidity associated with chronic disease.

Individual studies that have reported OHRQoL outcomes have indentified predictors of poor OHRQoL. These included disease severity, dental status, social class and cultural background. Unfortunately, there has been a lack of uniformity in methods used to collect these data, and this has created some difficulty in generalizing the results of individual studies. A variety of OHRQoL measures have been used, ranging from ad hoc, non-validated questionnaires (mostly used in the early nineteen nineties when quality of life was not a general used concept in dentistry yet), to comprehensive measures based on conceptual models and validated for use in particular populations. In the case of the latter measures, scoring systems have varied and been reported variously as prevalence, severity, and combinations of negative and positive perceptions of health. Finally, population studies have for the most part used shortened versions of validated measures such as the OHIP and this may lead to under-reporting of impacts.

Given these concerns, this review of the literature aimed to assimilate all of the available information on the relationship between tooth loss and OHRQoL in a systematic way using existing guidelines for conducting a systematic review. There were some limitations common to most systematic reviews, primarily difficulty in accessing literature not published in English. In order to minimize the possibility of publication bias, authors with acknowledged expertise in the field were contacted to determine if they had relevant data, which had not yet been published. They were also asked to clarify issues in their published research, which gave rise to uncertainty during the data extraction phase of the review. Accordingly, we believe that we have minimized the impact of reporting and publication bias.

Quality assessment of included studies was restricted to the use of exclusion criteria. These included minimal criteria of sample description (age and gender distribution) but not for example Socio Economic Status (SES). Other criteria indicating the quality of surveys, such as the number of observers, observer agreements, representativeness for larger samples, and the use of validated instruments were not always described, but were not used in the exclusion process. For instance, nine of the included studies were validation studies and these studies - presenting relevant data - would have been excluded in case the use of a validated instrument were an inclusion criterion. Although these studies were designed for another purpose, i.e. to test the psychometric properties of newly developed OHRQoL instruments, it was considered to be appropriate to use data on the number of missing teeth from these studies.

As far as we are aware of, this is the first systematic review and meta-analysis of the relationship between OHRQoL and tooth loss. Data from our systematic review and meta-analyses of observational studies provide fairly strong evidence that tooth loss is, on the whole, viewed negatively. This is a consistent finding, and appears to be independent of the OHRQoL measure used to assess subjective impact and context (e.g., country of residence). However, the severity of impairment of OHRQoL is probably context dependent [43]. Moreover, the severity of impairment might be associated with location and distribution of missing teeth, as suggested by the outcome of the meta-analysis of data of a Greek and a British population (Figure 5). Although associated, the correlation between number of missing teeth and number of occluding pairs (which is a derivative of the distribution of missing teeth) is not linear [54]. Therefore, the impact of cultural background, and location and distribution of missing teeth remains subject for further exploration.

It should be acknowledged that all studies are reported at population level, and this may mask heterogeneity of scores at an individual level. The latter is reflected by the wide variation in outcome scores in the meta-analyses as presented in Figures 2, 3, 4, 5, 6 and 7. Despite this, it seems that the negative view of tooth loss may ultimately result in demand for treatment to replace missing teeth. This will include a demand for dental implant retained restorations and other costly forms of treatment with a high burden of maintenance. Acceptance of dental extraction and replacement of teeth with conventional removable dentures, either partial or complete, has diminished [50]; furthermore, ability to adapt to complete replacement dentures in old age is also uncertain and best avoided if possible. This poses a considerable challenge for oral health care policy makers, and it is unlikely that all demand for high cost treatment interventions can be met solely by publicly funded healthcare.

The shortened dental arch concept has been described as means of providing sub-optimal, but acceptable level of oral function [55]. In limiting treatment goals to providing a shortened dental arch, costs of care can be minimized. The results of the review suggest that the number of occluding pairs of teeth is an important predictor of OHRQoL, and that the prevalence of negative impacts increases sharply once the number of teeth present drops below 20. It seems reasonable to suggest that application of the shortened dental arch approach is acceptable, particularly to older adults, and this may help inform public policy for oral health care in older age groups. The data also suggest that preventive strategies aimed at reducing tooth loss need to be reinforced. As reported by Petersen and Yamamoto [56], most oral diseases and chronic disease share common risk factors, and national health programs should incorporate disease prevention and health promotion using a common risk factor approach. Given the rising burden of chronic disease in an aging population, coupled with its negative impact on quality of life, this should receive urgent attention from policy makers.

## Conclusions

This study provides fairly strong evidence that tooth loss is associated with impairment in OHRQoL. This association appeared to be independent from the OHRQoL instrument used and context (e.g., cultural background) of the included samples. However, the extent and severity of impairment seems to be context dependent. Moreover, this study indicates that not only number, but also location and distribution of missing teeth affect the severity of OHQoL impairment. Given the negative consequences of tooth loss on OHRQoL, it is important that disease prevention measures are promoted when formulating health policy for older adults. It is likely that there will be greater demand from patients for treatment aimed at preserving teeth. The effectiveness of preventive strategies will require further research, and further economic analysis of tooth replacement strategies is also required.

## Competing interests

The authors declare that they have no competing interests.

## Authors' contributions

AEG designed the study, assessed all included publications for eligibility and drafted the manuscript. EMB performed the statistical analyses and assisted in the interpretation of the data and helped to draft the manuscript, PFA participated in the design of the study and assessment of the included papers and helped to draft the manuscript, DJW helped to draft the manuscript, NHJC participated in the design of the study, assessed all included publications for eligibility and helped to draft the manuscript. All authors read and approved the final manuscript.

## Supplementary Material

Additional file 1 Table S1: Summary of primary and additional outcomes of all included studies Click here for file

Additional file 2 **Summary of data of all included studies and feasibility for meta-analysis**. Click here for file
