# Supplementary material for: Tooth loss and oral health-related quality of life: a systematic review and meta-analysis
Source: Health Qual Life Outcomes. 2010 Nov 5;8:126. doi: 10.1186/1477-7525-8-126 (PMC2992503; doi:10.1186/1477-7525-8-126)
Supplement: Additional file 1 — Table S1: Summary of primary and additional outcomes of all included studies [file 1477-7525-8-126-S1.DOC]

### Additional file 1 – Summary of primary and additional outcomes of all included studies

^CS^ = cross-sectional study; ^LT^ = longitudinal study; ^CO^ = cohort study; ^VA^ = validation. ^1, 2^ same numbers indicate same sample.

| **First author, year** | **Population/sample**  **n (% females)** | **Subject of the study** | **Main outcomes concerning missing teeth** | **Other outcomes/conclusions** |
| --- | --- | --- | --- | --- |
| *OHIP-49 (Oral Health Impact Profile)* | | | | |
| Bae, 2007^VA^ [22] | Korean elderly  n =128 (51) | OHIP validation for elderly | Number of missing teeth associated with OHRQoL impairment. | Perceived treatment need also associated with OHRQoL impairment. |
| Hassel, 2006^CS^ [23] | German institutionalized elderly  n = 159 (81) | Dental and non-dental factors on OHRQoL of institutionalized elderly | No difference in OHRQoL between dentate and edentulous subject.s | Fewer teeth in static occlusion associated with OHRQoL impairment (bivariate analysis).  Non-dental factors had a striking effect on OHRQoL. The model with variables education, general pain status, denture retention, age of denture and kind of denture, explained 34% of the variance in OHIP total scores. |
| Ide, 2004^VA^ [12] | Japanese employees  n = 6079 (25) | OHIP validation for young and middle-aged adults | Number of missing teeth associated with OHRQoL impairment. | Outcome independent of gender, age, and denture wearing. |
| Locker, 1994^LT^ [29] | Canadian older adults  n = 312 (54) | Clinical and subjective indicators of oral health status and OHRQoL | Number of missing teeth associated with OHRQoL impairment. | Non-dental factors (general health, life stress, dental insurance, household income and age) explained almost as much variance in OHIP scores as the number of missing teeth (14, respectively 18%). |
| Mason, 2006^CO^ [30] | British middle aged adults  n = 281 (57) | Risk factors, lifecourse and OHRQoL | Number of retained teeth more impact on OHRQoL in woman; impact for men almost absent. | OHRQoL in men mostly explained by ‘early-life’ factors. |
| Walter, 2007^CS^ [48] | Canadian rural adults  n = 140 (64) | Clinical and socio-demographic variables and OHRQoL | Number of present natural teeth was not a significant variable in OHRQoL. | Missing anterior teeth associated with OHRQoL impairment.  OHRQoL positively related to molar pairs occluding, anterior pairs occluding, premolar pairs occluding and total number of occluding pairs.  Gender and normative endodontic treatment need also effected OHRQoL. |
| *OHIP-14 (Oral Health Impact Profile short version)* | | | | |
| de Oliveira, 2005^VA^ [39] | Brazilian postpartum women  n = 504 (100) | Validation of the Brazilian OHIP-14 | Tooth loss associated with OHRQoL impairmen.t | Perceived and normative dental treatment needs, self-rating oral health, pattern of dental attendance and untreated dental caries also associated with OHRQoL impairment. |
| Ekanayake, 2004^CS^ [10] | Sinhalese elderly  n = 235 (60) | Oral health status and oral impacts in elderly | Weak association between number of missing teeth and OHRQoL impairment. | Wearing dentures and halitosis were significant predictors of OHRQoL impairment. |
| Lahti, 2008^CS^ [25] | Finish adults aged over 30 yrs  n = 5897 (53) | Prevalence and severity of oral health impacts | Subjects with more missing teeth have higher prevalence of, and more severe impact on OHRQoL. | Subjects with 20 or more teeth wearing RPD more likely to report oral impacts than those with 20 or more teeth without RPD. Young subjects with low educational level had higher and more impacts. |
| Lawrence, 2008^CO^ [26] | 32-year old New Zealanders  n = 924 (49) | Dental and non-dental factors on OHRQoL in a birth cohort | Tooth loss associated with OHRQoL impairment. | Clinical oral health status indicators associated with OHRQoL impairment, independent of gender and SES. |
| Mariño, 2008^CS^ [4] | Australian older adult migrants  n = 603 (64) | Self-reported and clinically determined oral health status aspredictors of OHRQoL | Number of missing teeth associated with OHRQoL impairment. | Male subjects, subjects with fewer reported oral health treatment need and subjects that did not need to sip liquid to swallow food had less OHRQoL impairment. |
| Pallegredara, 2008^CS^ [40] | Sinhalese elderly  n = 630 (54) | Tooth loss, denture status and OHRQoL | Number of missing teeth most significant predictor of OHRQoL. | Anterior spaces more impact on OHRQoL than posterior spaces. |
| Steele, 2004^CS^ [43] | Australian and British adults  n = 3406 (59) (A)  n = 3662 (54) (B) | Age and tooth loss and OHRQoL | Number of missing teeth associated with OHRQoL impairment (A & B). | Age and denture wearing (A), and age, gender, and denture wearing (B) associated with OHRQoL impairment.  95% unexplained variance. |
| *GOHAI (Geriatric Oral Health Assessment Index)* | | | | |
| Atchinson, 1990^VA^ [19] | American elderly  n = 1755 (57) | Development and validation of GOHAI | Number of missing teeth associated with OHRQoL impairment. | The non-dental variables being male, being white, well-educated, higher income was associated with better OHRQoL. |
| Mesas, 2008^CS^ [37] | Brazilian urban elderly  n = 267 (60) | Dental and non-dental factors on OHRQoL | Edentulism effected OHRQoL (one out of three GOHAI dimensions). | Absence of posterior occlusion effected the physical dimension; gender and depression associated with OHRQoL impairment; no association with: age, schooling, SES and medication. |
| Naito, 2006^VA^ [38] | Japanese elderly  n = 175 (68) | Validation of the Japanese GOHAI | Number of missing teeth associated with OHRQoL impairment. | Periodontal condition not associated with impaired OHRQoL; wearing RPD effected OHRQoL negatively. Non-clinical factors (low level of education, and perceived poor oral health, poor health and dental care needs) associated with OHRQoL impairment. |
| Swoboda, 2006^CS^ [44] | American low income elderly  n = 733 (56) | Dental and non-dental predictors on OHRQoL | Number of missing teeth associated with OHRQoL impairment. | Functional dentition was a less significant predictor than ethnicity and being foreign-born. Together with gender, years since immigration and number of carious roots and periodontal status these variables predicted 32% of the variance. |
| Tubert-Jeanin 2003^VA^ [46] | Economically disadvantaged French adults  n= 260 (49) | Validation of French GOHAI | Number of missing teeth associated with OHRQoL impairment. | Number of decayed teeth, filled teeth and presence of RPD associated with OHRQoL impairment. |
| Tubert-Jeannin 2004, ^CS^ [47] | Subsample of Tuber-Jeannin 2003  n = 129 (55) | Dental status and OHRQoL | Number of missing teeth associated with OHRQoL impairment. | Number of decayed teeth associated with OHRQoL impairment; no association with: filled teeth, exposed roots and plaque index. |
| Wong, 2005^CS^ [49] | Non-institutionalized Elderly in Hong Kong  n = 233 (73) | Toots loss, denture wearing and OHRQoL | No association between number of natural teeth present and OHRQoL. | Subjects with fewer than 20 teeth had impaired OHRQoL compared to subjects with 20 or more teeth.  Number of loose teeth, difficulty in accepting tooth loss and satisfaction with denture explained 25% of the variance in GOHAI score. |
| *OIDP (Oral Impact on Daily Performance)* | | | | |
| Kida, 2006^VA^ [24] | Tanzanian older adults  n = 1020 (54) | Validation of Kiswahili OIDP | In rural subjects the number of missing teeth was associated with OHRQoL impairment; in urban subjects this association was not present. | Toothache and loose teeth were the most frequently perceived cause of impairments of daily performances. |
| Sheiham, 2001^CS^ [41] | British institutionalized and non-institutionalized elderly  n = 798 (57) | Prevalence of dental impacts and their effects on eating | In non-institutionalized subjects more missing teeth associated with daily impacts, indicating OHRQoL impairment. | Being edentulous is associated with more impacts in non-institutionalized elderly but with fewer impacts in institutionalized elderly. |
| Tsakos, 2006^CS^ [5] | British non-institutionalized elderly  (subsample of Sheiham, 2001)  n = 736 (48) | Clinical correlates of OHRQoL | No relationship between the number of teeth and the prevalence of oral impact. | Of the clinical variables in the model (decayed, filled or mobile teeth, unfilled anterior spaces, NOPs and POPs and AOPs) only NOPs and AOPs were statistically significant associated with daily impacts. |
| Tsakos, 2004^CS^ [45] | Greek non-institutionalized elderly  n = 448 (64) | Relationship between clinical dental measures and OHRQoL | Number of missing teeth associated with daily impacts, indicating OHRQoL impairment. | Of the clinical variables in the model (decayed, filled or mobile teeth, unfilled anterior spaces NOPs and POPs) only filled teeth, unfilled anterior spaces, fewer NOPs and fewer POPs were statistically significant associated with daily impacts. |
| *OHQoL-UK (UK oral health related quality of life measure)* | | | | |
| McGrath, 2001^VA^ [33] | British adults  n= 390 (58) | Validation of the OHQoL-UK(W) | Number of missing teeth associated with “bad effect on QoL” indicating OHRQoL impairment. | The tested variables (older age, being employed, having Asian background and having no denture) were all associated with “good effect on QoL”. |
| McGrath, 2001^CS^ [32] | British adults  n = 1801 (55)^1^ | Variations in impact of OHRQoL in relation to number of teeth and denture status | Number of missing teeth associated with “bad effect on QoL” indicating OHRQoL impairment. | Subjects with less than 20 teeth who did not have recourse to a denture had poor OHRQoL. |
| McGrath, 2002^CS^ [34] | British adults  n = 1838 (55)^2^ | Establishment of normative age-gender values for OHQoL-UK | Subject with less than 20 teeth were more likely to have reduced OHRQoL compared to those having 20 or more teeth. | Younger ages and lower social class were more likely to have reduced OHRQoL. Gender did not influence the outcome. |
| McGrath, 2004^VA^ [35] | British adults  weighted sample  n = 1801 (55)^1^  unweighted sample  n = 1838 (55)^2^ | Value of self-weighting OHRQoL items in assessing OHRQoL | No additional outcomes in relation tooth loss to McGrath 2001 and McGrath 2002. | No additional outcomes to McGrath 2001 and McGrath 2002. |
| McGrath, 2003^VA^ [31] | Syrian adults  n = 369 (37)  Egyptian adults  n = 292 (36)  Saudi Arabian adults  n = 284 (46) | Validation of the Arabic version of the OHQoL-UK | For all three samples, subjects with 20 or more teeth reporting not experiencing any oral health problem in the past had higher OHQoL-UK scores than their counterparts, indicating better OHRQoL. | For all three samples, higher OHQoL-UK score associated with higher education and higher SES. |
| *DIDL (Dental Impact of Daily Living)* | | | | |
| Leao 1995^CS^ [28] | Brazilian adults  n = 662 (46) | Comparison of subjective impact dimensions with clinical dental status | Number of missing teeth associated with impacts on daily living, indicating OHRQoL impairment. | Social class and gender explained 3% of the variance. When DMFT was added the explained variance increased to 19%. After adding periodontal variables the explained variance was 24%. |
| *Custom-made satisfaction questionnaires* | | | | |
| Steele, 1997^CS^ [42] | British dentate elderly  n = 1211 (52) | Clinical factors related to reported satisfaction with oral function | Having fewer than 20 teeth associated with dissatisfaction, indicating OHRQoL impairment. | Complaints with appearance, unfilled anterior spaces, dry mouth, eating hard foods, limitation of food selection and speech were associated with dissatisfaction. |
