# Supplementary material for: Tooth loss and oral health-related quality of life: a systematic review and meta-analysis
Source: Health Qual Life Outcomes. 2010 Nov 5;8:126. doi: 10.1186/1477-7525-8-126 (PMC2992503; doi:10.1186/1477-7525-8-126)
Supplement: Additional file 2 — Summary of data of all included studies and feasibility for meta-analysis. [file 1477-7525-8-126-S2.DOC]

### Additional file 2 – Summary of data of all included studies and feasibility for meta-analysis

1: no study included with comparable categorization of subjects; 2: no study included with comparable outcome measure; 3: no study included in which comparable OHRQoL instrument was used.

| **First author, year** | **Country** | **Study design** | **Population** | **Categorization** | **Outcome measure** | **Results** | **Unadjusted data presented +/-** | **Deviation presented (e.g. SD, SE, 95%CI) +/-** | **meta-analysis feasible yes/no (reason)** |
| --- | --- | --- | --- | --- | --- | --- | --- | --- | --- |
| *OHIP-49 (Oral Health impact profile)* | | | | |  |  |  |  |  |
| Baba, 2008a | Japan | multicenter cross-sectional | adults with shortened dental arches  n = 115 (70),  mean age (SD): 58.6 (10.0) | group 1:  two missing POPs in SDA  group 2:  four missing POPs in SDA  group 3:  five or six missing POPs in SDA | mean scores (95% CI) | group 1: 26.3 (16.5 - 36.1)  group 2: 35 (21.9-48.2)  group 3: 40.8 (26.2-55.3) | + | + | no (1) |
|  |  |  |  |  | cor. coef (95% CI)  dependent variable: total score | group 1 vs. group 2 + 3: 11.1 (2.08-19.2)  p = 0.02  constant: 26.3 (16.5 – 36.1) t = 6.9  p = 0.00 | - |  | no |
| Baba, 2008b | Japan | multicenter cross-sectional | adults with shortened dental arches  same sample as Baba, 2008a  n = 115 (70)  mean age (SD): 58.6 (10.0) | posterior occlusal units (OUs) in SDAs | cor. coef (95% CI) (regression analysis)  dependent variable: total score | each missing POP was associated with an increase of 2.1 (0.6-3.5) OHIP-J units  p = 0.012 | - |  | no |
| Bae, 2007 | Korea | validation study | elderly aged ≥ 54 years  n = 128 (51)  mean age (SD): 65.2 (7.2) | no. of present teeth | Pearson’s cor. coef.  dependent variable: total score | no. of present teeth:  -0.44 p < 0.001 | - | - |  |
| Hassel, 2006 | Germany | cross-sectional survey | institutionalized elderly  n = 159 (81)  mean age (range): 82.8 (61-98) | less teeth in static occlusion  dentate/edentate | median scores (SD) (bivariate testing) | less teeth in static occlusion:  higher OHIP score (numbers not presented):  significance p< 0.004  dentate/edentate :  edentate: 31 (24)  dentate: 28 (25)  p = 0.44 | - |  | no |
|  |  |  |  |  |  |  | + | + | no (2) |
| Ide, 2004 | Japan | cross-sectional survey | employees  n= 6079 (25)  age composition:  20-29yrs n = 1272  30-39yrs n = 1358  40-49yrs n = 1921  50-59yrs n = 1528 | no. of missing teeth: 0, 1-4, 5-28 | mean score (no SD presented) | no. of missing teeth:  0: 30.5  1-4: 39.4  5-28: 54.9 | + | - | no |
|  |  |  |  |  | standardized partial regression. coef. (multiple linear regression)  dependent variable: total score | no. of missing teeth:  0.247 p < 0.001 | - |  | no |
| Locker, 1994 | Canada | longitudinal epidemiological study | older adults aged ≥50 years  n = 312 (54)  mean age: 63 | dentate/edentate | mean score (SD) | dentate: 40.2 (35.9)  edentate: 78.7 (63.6)  p <0.001 | + | + | no (1) |
|  |  |  |  | no. of missing teeth | beta (regression analysis)  dependent variable: square root of OHIP score | no. of missing teeth:  0.35 p < 0.0001 | - | - | no |
|  |  |  |  | no. of missing teeth, no. of present functional units,  no. of present posterior functional units  (incl. edentate subjects) | cor. coef.  dependent variable: total score | no. of missing teeth:  0.43 p < 0.001  no. of present functional units :  -0.42 p <0.001  no. of present posterior functional units :  -0.42 p < 0.001 | - |  | no |
| Mason, 2006 | U.K. | cohort study | cohort of subjects aged 49-51  n = 281 (57)  age: 49-51 | no. of present teeth | % contribution to explaining the variation in total score | % contribution to explaining the variation in total score :  men: 1.4  woman: 15.9 | - |  | no |
|  |  |  |  |  | % contribution to explaining the variation number of impacts | % contribution to explaining the variation number of impacts:  men: 0.5  women: 8.9 |  |  |  |
| Walter, 2007 | Canada | cross-sectional survey | adults aged ≥ 18 years living in a rural environment  n = 140 (64)  age composition:  18-24: 24%  25-34: 31%  35-44: 25%  45-54: 11%  55-64: 5%  65-74: 4%  75-84: 1% | anterior teeth missing:  yes/no | OR (95% CI) (multivariable regression analysis)  dependent variable: OHIP total < 75^th^ /≥ 75^th^ percentile | anterior teeth missing yes/no:  21.5 (7.1-75.7)  p < 0.001 | - |  | no |
| OHIP-14 (Oral Health Impact Profile short version) | | | |  |  |  |  |  |  |
| deOlveira, 2005 | Brazil | cross-sectional survey | postpartum woman  n = 504 (100);  mean age (SD): 24 (6.2) | missing teeth  yes/no | mean scores (SD) | missing teeth:  yes: 9.0 (11.4);  no: 5.9 (8.9)  p = < 0.05 | + | + | no (1) |
| Ekanayake, 2004 | Sri Lanka | cross-sectional survey | elderly aged ≥ 60 years  n = 235 (60)  dentate sample n = 171  41% in 70-79 age group | no. of missing teeth:  0-11, 12-31 | mean rank (bivariate testing) | no. of missing teeth:  0-11: 76.61  12-31: 91.47  p = 0.02 | + | - | no |
|  |  |  |  | no. of missing teeth: | cor. coef. (Spearman rank)  dependent variable: total score | no. of missing teeth:  0.18  p = 0.021 |  |  |  |
| Lahti, 2008 | Finland | cross sectional survey | adults aged ≥ 30 years  n = 5897 (53)  age composition:  30-34yrs n = 665 35-44yrs n = 1387 45-54yrs n = 1537 55-64yrs n = 1032  65-74yrs n = 713  75-84yrs n = 421  85+ yrs n = 142 | no. of present teeth: 0, 1-8, 9-16, 17-20, 21-24, 25-32 | mean scores (95% CI) | see meta-analysis | + | + | yes |
|  |  |  |  |  | % of subjects reporting at least one impact | see meta-analysis | + | + | yes |
|  |  |  |  | no. of present teeth: 0, 1-19 | OR (95% CI)  dependent variable: having impact yes/no | see meta-analysis | + | + | yes |
| Lawrence, 2008 | New Zealand | birth cohort study | cohort of dentate subjects of 32-year old  n = 924 (49)  age: 32 years | no. of missing teeth:  0, 1+ | OR  dependent variable: having impact yes/no | see meta-analysis | + | + | yes |
|  |  |  |  |  | cor. coef. (IRR (95% CI)) (poisson regression model)  dependent variable: extent: prevalence of impacts  severity: mean total score | extent: 0.12 (1.12 (1.09-1.15))  p = <0.001  severity: 0.06 (1.07 (1.06-1.08))  p = <0.001 | - |  | no |
| Mariño, 2008 | Australia | cross-sectional survey | dentate older migrant adults 55 years and over from Greek and Italian backgrounds  n = 603 (64)  mean age (SD): 67.7 (6.2) | no. of missing teeth | beta (SD) (multiple regression)  dependent variable: total score | no. of missing teeth:  0.24 (0.05)  p = 0.0001 | - |  | no |
| Pallegedara, 2008 | Sri Lanka | cross sectional survey | free living older adults aged ≥ 60 years  n = 630 (54)  mean age (SD): 68.9 (7.1) | non-denture wearers :  no. of missing teeth:  1-8, 9-16, 17-24, 25-31  anterior spaces:  yes/no  no. of missing posterior teeth:  1-12, 13-20 | median impact score (IQR) | no. of missing teeth:  1-8: 0 (0.0, 2.0)  9-16: 2 (0.0, 6.0)  17-24: 6 (3.0, 9.0)  25-31: 6 (3.0, 10.0)  p = 0.0001  anterior spaces:  yes: 4 (0.0, 8.0)  no: 0 (0.0, 2.0)  p = 0.0001  no. of missing posterior teeth:  1-12: 1.5 (0.0, 4.0)  13-20: 6.0 (2.0, 9.0)  p = 0.0001 | + | + | no (2) |
|  |  |  |  |  | OR (95% CI)  (multiple logistic regression)  dependent variable: total score ≤2/>2 | no. of missing teeth:  1-8: 1.00  9-16: 2.13 (1.24-3.66) p = 0.006  17-24: 3.51 (1.51-8.13) p = 0.003  25-31: 4.21 (1.58-11.19) p = 0.004  anterior spaces:  no: 1.00  yes: 2.76 (1.62-4.72)  p = 0.001  no. of missing posterior teeth:  1-12: 1.00  13-20: 1.23 (0.64-2.37)  p = 0.52 | - |  | no |
| Steele, 2004 | UK/Australia | two national surveys | adults aged ≥ 16 years  Australian sample:  n = 3406 (59)  age composition:  <30: 14.4%  30-49: 40.7%  50-69: 33.6%  70+: 10.4%  59% f  UK sample:  n = 3662 (54)  age composition:  <30: 19.7%  30-49: 44.5%  50-69: 28.3%  70+: 7.4% | no. of present teeth: 1-8, 9-16, 17-20, 21-24, 25-32 | mean scores (SE) | see meta-analysis | + | + | yes |
| GOHAI (Geriatric Oral Health Assessment Index | | | | |  |  |  |  |  |
| Atchinson, 1990 | USA | validation study | convenience sample of older adults  n = 1755 (57)  mean age: 74 (5.6) | no. of present teeth | cor. coef.  (Pearson)  dependent variable:  total score | no. of present teeth:  0.33 p = 0.001 | - |  | no |
| Mesas, 2008 | Brazil | cross sectional survey | adults aged between 60-74 years living in the urban zone of a city in Brazil  n= 267 (60)  mean age (SD): 66.5 (4.1) | edentulism:  yes/no  absence of posterior occlusion:  yes/no | mean scores (SD) per dimension | edentulism:  physical dimension:  yes: 10.76 (1.83)  no: 11.29 (1.20)  p = 0.0421  social and worry dimension: N.S. (p = 0.27 and 0.73 respectively)  absence of posterior occlusion  physical dimension:  yes: 10.54 (1.87)  no: 11.25 (1.48)  p = 0.0038  social and worry dimension: N.S. (p = 0.31 and 0.82 respectively) | + | + | no (1) |
| Naito, 2006 | Japan | survey (validation study) | adults aged ≥ 60 years  n = 175 (68)  mean age 70.0 (6.4) | no. of present teeth:  0, 1-19, 20+ | mean scores (SD) | no. of present teeth:  0: 38.7 (18.5);  1-19: 46.3 (9.7)  20+: 54.0 (8.8)  p = < 0.001 | + | + | yes |
| Swoboda, 2006 | USA | cross-sectional study | low income elderly aged 60–75 years with at least 4 remaining teeth  n = 733(56)  mean age 72.7 (4.7) | no. of present teeth  no. of occluding pairs  no. of molar occluding pairs  no. of anterior occluding pairs  no. of premolar occluding pairs | cor. coef. (Spearman)  dependent variable:  total score | no. of present teeth:  0.21 p =0.0001  no. of occluding pairs:  0.26 p =0.0001  no. of molar occluding pairs:  0.23 p =0.0001  no. of anterior occluding pairs:  0.22 p =0.0001  no. of premolar occluding pairs:  0.21 p =0.0001 | - |  | no |
| Tubert-Jeannin, 2003 | France | validation study | low income persons benefiting from the national health insurance system  n = 260 (49)  age composition:  18-45yrs, 65% being 30yrs or older | no. of present teeth: 0-19, 20+  no. of missing teeth: 0-3, 4+ | mean scores (SD) | no. of present teeth:  0-19: 36.12 (8.58)  20+: 47.81 (8.76)  p < 0.001  no. of missing teeth:  0-3: 48.72 (8.46)  4+: 41.38 (9.74)  p < 0.001 | + | + | yes |
| Tubert-Jeannin, 2004 | France | survey | adults aged 35-44 years with low income  subsample of Tubert-Jeannin, 2004:  n = 129 (55)  age: 35-44 years | no. of present teeth  no of missing teeth not replaced | OR (95%CI) (multiple logistic regression)  dependent variable: total score | no. of present teeth:  1.09 (0.99-1.19)  no of missing teeth not replaced:  0.85 (0.70-1.02) | - |  | no |
| Wong,2005 | China  (Hong Kong) | survey | community dwelling elderly aged 60-80 years  n = 233 (73)  age composition:  60-69yrs: n= 61  70-79yrs: n = 172 | edentulous  partially dentate with RPD  partially dentate without RPD | mean score (SD) | edentulous:  53.0 (6.3)  partially dentate with RPD:  49.1 (7.2)  partially dentate without RPD:  50.8 (6.7)  p = 0.001 | + | + | no (1) |
| OIDP (Oral Impact on Daily Performance) | | | |  |  |  |  |  |  |
| Kida, 2006 | Tanzania | cross-sectional survey | non-institutionalized Tanzanians adults aged ≥ 50 years  n = 1020 (54)  mean age (SD): 62.9 (10.6) | no of missing teeth: 0-10, 11-19, 20+ | OR (95% CI)  dependent variable:  having impact yes/no | see meta-analysis | + | + | yes |
| Sheiham, 2001 | U.K. | national survey | non-institutionalized and institutionalized adults aged ≥65 years  n = 955 (initial sample) (55) (weighted base)  dentate sample: n = 395  age composition (weighted base):  65-74: n= 423  75-84: n = 195  85+: n = 46 | no of present teeth: 0-10, 11-20, 21+ | % participants with impact | see Tsakos, 2006 | + | + | no |
|  |  |  |  |  | OR (95% CI) adjusted by age, gender and education level  dependent variable:  having impact yes/no | no. of present teeth:  1-10: 1  11-20: 0.50 (0.23-1.08) p = 0.08  21+: 0.35 (0.14-0.90)  p = 0.03 | - |  | yes |
| Tsakos, 2006 | UK | national survey | non-institutionalized adults aged ≥65 years  subsample of Sheiham, 2001  n = 736 (48)  dentate sample: n = 399  age:  56.9% ≥ 75 years | no of present teeth:  1-10, 11-20, 21+  unfilled anterior spaces:  no/yes  no. of present natural occluding pairs present:  0-8, 9-16;  no. of present anterior occluding pairs present:  0-2, 3-6  no. of present posterior occluding pairs present:  0-3, 4-8 | % participants with impact | see meta-analysis | + | + | yes |
|  |  |  |  |  | OR (95% CI) adjusted by age, gender and educational level  dependent variable:  having impact yes/no | no. of present teeth:  1-10: 2.02 (0.86-4.71) p= 0.104  11-20: 1.44 (0.61-3.35) p = 0.397  21+: 1  unfilled anterior spaces  no: 1  yes: 1.15 (0.54-2.44) p < 0.707  no. of present natural occluding pairs:  0-8: 2.66 (1.08-6.51) p = 0.032  9-16: 1  no. of present anterior occluding pairs present:  0-2: 3 (1.55-5.81) p = 0.001  3-6: 1  no. of present posterior occluding pairs present:  0-3: 1.82 (0.84-3.67) p = 0.126  4-10: 1 |  |  |  |
| Tsakos, 2004 | Greece | cross-sectional survey | non-institutionalized adults aged ≥65 years  n = 448 (64),  mean age: 71.4 (6.9) | no of present teeth: 0-10, 11-19, 20+ | % participants with impact | see meta-analysis | + | n.a. | yes |
|  |  |  |  | no of present teeth:  1-10, 11-20, 21+  unfilled anterior spaces:  no/yes  no. of present natural occluding pairs present:  0-8, 9-16;  no. of present posterior occluding pairs present:  0-3, 4-8 | OR (95% CI) adjusted by age group, sex and educational level  dependent variable:  having impact yes/no | no. of present teeth:  1-10: 2.05 (1.25-3.35) p= 0.004  11-20: 1.85 (1.11-2.95) p = 0.016  21+: 1  unfilled anterior spaces  no: 1  yes: 2.86 (1.70-4.0) p < 0.001  no. of present natural occluding pairs:  0-8: 1.72 (1.14-2.58) 1 p = 0.009  9-16:  no. of present posterior occluding pairs present :  0-3: 1.57 (1.04-2.36) p = 0.031  4-8: 1 | - |  | no |
| **OHQoL-UK (Oral Health Quality of Life UK)** | | | |  |  |  |  |  |  |
| McGrath, 2001 | United Kingdom | validation study | convenience sample of adults  n = 390 (58)  age composition:  18-24: n = 62  25-34: n = 82  35-44: n = 76  45-54: n = 90  55-64: n = 35  65-74: n = 25  75+: n = 20 | number of teeth present:  > 20, 10-19, < 10 | median score (IQR) | number of teeth present:  > 20: 91 (80-102)  10-19: 84 (77-91)  < 10: 80 (75-83)  p<0.01 | + | + | no (1) |
| McGrath, 2001 | United Kingdom |  | Adults  n = 1801 (55)  age categorization:  16-64: n = 1384  ≥ 65: n= 417 | no. of present teeth: 0-19, 20+ | weighted means scores (SD) | no. of present teeth:  0-19: 85 (14)  20+: 92 (17);  p < 0.001 | + | + | no (3) |
| McGrath, 2002 | United Kingdom |  | Adults  n =1838 (55)  age composition:  16-24: n = 179  25-34: n = 355  35-44: n = 343  45-54: n = 290  55-64: n = 255  65-74: n = 224  75+: n = 192 | no. of present teeth: 0-19, 20+ | OR (95% CI) controlling for other factors  dependent variable:  0 = median score or above, 1 = below median score | 2.12 (1.17-1.73)  p <0.05 | - |  | no |
|  |  |  |  |  | regression coef.(S.E.) | 0.75 (0.13) p <0.05 |  |  |  |
| McGrath, 2004 | United Kingdom |  | Adults  same sample as McGrath, 2001  weighted sample  n = 1801 (55)  age categorization:  16-64: n = 1384  ≥ 65: n= 417  same sample as McGrath, 2002  unweighted sample  n =1838 (55)  age categorization:  16-64: n = 1422  ≥ 65: n= 416 | no. of present teeth: 0-19, 20+ | weighted and unweighted means scores (SD) | weighted scores:  no. of present teeth:  see McGrath, 2001 | + | + | no (1) |
|  |  |  |  |  |  | unweighted scores:  no. of present teeth:  0-19: 52.3 (8.9)  20+: 56.9 (10.2)  p < 0.01 | + | + | yes |
| McGrath[5], 2003 | Syria, Egypt and Saudi Arabia | validation study | convenience samples | no. of present teeth: 0-19, 20+ | unweighted means scores (SD) | no. of present teeth:  Syria:  20+: 60 (10);  0-19: 56 (9);  Egypt:  20+: 56 (9);  0-19: 53 (8);  Saudi Arabi:  20+: 62 (10);  0-19: 57 (8)  p < 0.01 | + | + | yes |
| **DIDL (Dental Impact of Daily Living)** | | | | |  |  |  |  |  |
| Leao, 1995 | Brazil | validation study | convenience sample of adults aged 35-44 years  n = 662 (46)  age: 35-44yrs | no. of missing teeth | cor. coef (Spearman) | no. of missing teeth:  -0.18 p = < 0.001 | - |  | no |
|  |  |  |  |  | beta (regression analysis)  dependent variable: total score | no. of missing teeth:  -0.28 p <0.0001 | - |  | no |
| **Custom-made satisfaction questionnaires** | | | | |  |  |  |  |  |
| Meeuwissen, 1995 | The Nether-lands | cross sectional survey | dentate non-institutionalized elderly  n = 320  age 55-59yrs n = 111, 60-64yrs, n = 130, 65-74 n= 88, 59F | no. of present occlusal units:  none, few, moderate, complete | average satisfaction scores | no. of present occlusal units:  none: 6.3  few: 10.3  moderate: 11.4  complete dentition: 14.0 | + | - | no |
| Steele, 1997 | U.K. | survey | adults aged ≥ 60 years  n = 1211 (52)  mean age: 68.1 | no. of present teeth:  < 21, 20+ teeth (with and without denture) | OR (95% CI)  dependent variable: overall satisfaction score | 3.2 (1.4-7.1) |  |  | no (3) |
| Leake, 1994 | Canada | longitudinal epidemiological study | partially dentate older adults (≥ 50yrs) with complete anterior dentitions  n = 338 (55)  mean age: 59.9 | no. of natural functional units present:  8, 5-7, 3-4, 0-2 | % subjects with impact | no. of natural functional units present:  8: 48  5-7: 58  3-4: 60  0-2: 51  p = 0.47 | + | - | no |
